# Upregulation of cellular exhaustion-related genes and CD4 + T cell disturbances in immunological non-responders on antiretroviral therapy

**DOI:** 10.1007/s12026-026-09859-y

**Published:** 2026-10-09

**Authors:** Henrique Fernando Lopes-Araujo, Wlisses Henrique Veloso Carvalho-Silva, José Leandro Andrade-Santos, Maria Carolina Santos Guedes, Fabrício Oliveira Souto, Luiz Cláudio Arraes De Alencar, Igor de Farias Domingos, Paulo Sérgio Ramos de Araújo, Kleyverson Feliciano-Santos, Rafael Lima Guimarães

**Affiliations:** 1 Department of Genetics, Federal University of Pernambuco - UFPE, Av. Prof. Moraes Rego, 1235, Cidade Universitária, Recife, Pernambuco 50670-901 Brazil; 2 Keizo Asami Institute (iLIKA), Federal University of Pernambuco - UFPE, Recife, Pernambuco 50670-901 Brazil; 3 Department of Immunology, Aggeu Magalhães Institute (IAM) – Oswaldo Cruz Foundation (FIOCRUZ), Recife, Pernambuco 50740-465 Brazil; 4 FACOL University Center - UNIFACOL, Vitória de Santo Antão, Pernambuco 55612-285 Brazil; 5 Institute of Integral Medicine Professor Fernando Figueira (IMIP), Recife, Pernambuco 50070-550 Brazil; 6 Hospital Correia Picanço (HCP), Recife, Pernambuco 52050-150 Brazil; 7 Hospital das Clínicas (HC-UFPE), Federal University of Pernambuco – UFPE, Recife, Pernambuco 50670- 901 Brazil; 8 Department of Tropical Medicine, Federal University of Pernambuco – UFPE, Recife, Pernambuco 50670- 901 Brazil; 9 Department of Parasitology, Aggeu Magalhães Institute (IAM) – Oswaldo Cruz Foundation (FIOCRUZ), Recife, Pernambuco 50740-465 Brazil

**Keywords:** AIDS, HIV, PDCD1, TIGIT, HAVCR2

## Abstract

Antiretroviral therapy suppresses HIV replication, but 10–40% of people living with HIV do not reconstitute CD4 + T cell counts despite viral suppression. Cellular exhaustion and alteration in the frequency of CD4 + T cell subsets are mechanisms involved in immunological nonresponse. This study investigated cellular exhaustion through gene expression and frequencies of CD4 + T cell subpopulations through flow cytometry in 36 male individuals living with HIV (28 immunological responders and 8 immunological non-responders) after 18 months of treatment. Gene expression analysis revealed significantly upregulation of *PDCD1* (fold change = 1.81; *p* = 0.0003) and *TIGIT* (fold change = 1.80; *p* = 0.0055) in immunological non-responders compared to immunological responders, with ROC curve analysis confirming discriminatory ability. Flow cytometry demonstrated lower frequencies of recent thymic emigrant (19.42 ± 7.76 vs. 32.29 ± 10.68; *p* = 0.0285) and naive CD4 + T cells (26.10 ± 9.41 vs. 47.65 ± 13.45; *p* = 0.0194) in immunological non-responders compared to immunological responders. Moreover, we observed higher frequency of central memory CD4 + T cells (47.94 ± 13.42 vs. 32.58 ± 9.89; *p* = 0.0184), with excellent discriminatory capacity. *TIGIT* expression inversely correlated with recent thymic emigrant (*r* = -0.7284; *p* = 0.0013) and naive CD4 + T cells (*r* = -0.7394; *p* = 0.0010), and directly with central memory CD4 + T cells (*r* = 0.7264; *p* = 0.0014). These findings emphasize cellular exhaustion and dysregulated CD4 + T cell subsets as critical factors of immunological nonresponse.

**Supplementary Information:**

The online version contains supplementary material available at https://doi.org/10.1007/s12026-026-09859-y.

## Introduction

Untreated infection by the human immunodeficiency virus (HIV) usually leads to progressive destruction of immune cells, especially CD4 + T cells [1, 2]. The final stage of HIV infection is acquired immunodeficiency syndrome (AIDS), in which people living with HIV (PLHIV) become profoundly immunocompromised and more susceptible to opportunistic infections and HIV-related complications, thereby increasing morbidity and mortality [3, 4]. The current treatment for PLHIV is the antiretroviral therapy (ART), which aims to suppress viral replication and reduce viremia to undetectable and untransmittable levels (< 50 copies of viral RNA/mL) [5, 6].

ART-treated PLHIV usually undergoes immune reconstitution through a gradual recovery of CD4 + T cells, as circulating virus levels are significantly reduced, preventing further destruction of these lymphocytes [7, 8]. PLHIV who achieve adequate immune reconstitution after viral suppression are defined as immunological responders (IR). However, approximately 10–40% of PLHIV under sustained viral suppression do not achieve satisfactory immune recovery and are classified as immunological non-responders (INR) [9, 10].

Immunological nonresponse is a multifactorial condition and may be influenced by several factors, for example, age, sex, time to ART initiation, and therapeutic regimen [11]. Underlying mechanisms contributing to immunological nonresponse may include insufficient production and/or elevated destruction of CD4 + T cells, which compromise the reconstitution of these lymphocytes, despite sustained virological suppression [12]. PLHIV are also prone to coinfections, microbial translocation, and residual viral replication within viral reservoirs [13]. These factors contribute to persistent and exacerbated immune activation, which may cause cellular exhaustion [14, 15].

T cell exhaustion occurs primarily due to persistent and prolonged antigenic stimulation during HIV infection. The cellular exhaustion results in a progressive loss of effector functions and increased expression of immune checkpoint receptors (ICR) on the cell surface [16]. This process may quantitatively contribute to immunological nonresponse, as exhausted cells exhibit increased susceptibility to apoptosis, alongside a reduced proliferative capacity and impaired secretion of IL-2, which is essential for CD4 + T cell renewal [17]. Functionally, exhausted cells exhibit a decline in their effector capacity, diminishing the coordination and effectiveness of immune responses that may contribute to immunological nonresponse even under sustained viral suppression. Although T cell exhaustion was first described in CD8 + T cells, CD4 + T cells are also susceptible to this phenomenon [18, 19]. Recent evidence indicates that INR exhibit higher levels of ICR than IR, suggesting that cellular exhaustion is associated with immunological nonresponse [20, 21].The hallmark of T cell exhaustion during HIV infection is the increased surface expression of ICR, for example, Programmed Cell Death Protein 1 (PD-1), T-Cell Immunoglobulin and ITIM Domain (TIGIT) and T-cell Immunoglobulin and Mucin Domain-Containing 3 (TIM-3) [22]. These receptors are encoded, respectively, by the *PDCD1*, *TIGIT*, and *HAVCR2* genes [23].

Therefore, this study sought to elucidate how the expression of genes related to cellular exhaustion could influence immunological nonresponse by comparing the gene expression levels between IR and INR groups. Clinical variables and CD4 + T cell subsets were compared to characterize the clinical profile and the frequency of these subpopulations between groups. Additionally, correlations were performed between gene expression and CD4 + T cell subsets to provide deeper insights into cellular exhaustion and immune reconstitution in ART-treated PLHIV.

## Materials and methods

### Study design and ethical considerations

Male ART-treated PLHIV were recruited from three referral centers in Pernambuco, Northeast Brazil: Hospital das Clínicas (HC-UFPE), Hospital Correia Picanço (HCP), and Institute of Integral Medicine Professor Fernando Figueira (IMIP). Eligible participants were male adults (≥ 18 years) who had been on ART for 18 months, demonstrated consistent adherence to treatment, and maintained viral suppression (< 50 copies of viral RNA/mL). Exclusion criteria were female individuals, history of intravenous drug use, coinfections during ART, autoimmune diseases, cancers, or other immune disorders. Medical records were reviewed to verify eligibility and collect clinical data. All participants provided written informed consent documents, confirming their voluntary participation and authorizing peripheral blood samples collection (9 mL in heparinized tubes). All collected samples were processed at the Keizo Asami Institute. The study was approved by the Ethics Committees of all participating hospitals: HCP (protocol number: 77566023.5.0000.5190), HC-UFPE and IMIP (protocol number: 65468222.3.1001.8807).

### Immunological classification

Participants were categorized as IR or INR based on CD4 + T cell counts and gains after 18 months of ART. IR were individuals who either initiated ART with or achieved and sustained a CD4 + T cell count ≥ 500 cells/µL within 18 months or gained ≥ 200 CD4 + T cells/µL during this period. INR were those with CD4 + T cell counts remaining below 500 cells/µL and gains < 200 cells/µL after 18 months of treatment. This classification was adapted from the criteria established by Cenderello et al. (2016) [24].

### Isolation of peripheral blood mononuclear cells

Peripheral blood mononuclear cells (PBMC) were isolated from blood samples through Ficoll-Paque Plus density gradient centrifugation and washed twice with 1x PBS. The cells were then resuspended in FACS buffer (3% BSA, 0.01% sodium azide, and PBS 1x).

### RNA extraction and cDNA synthesis

RNA was isolated from PBMC using the Trizol-chloroform method (Invitrogen), following the manufacturer’s protocol. RNA concentration and purity were measured using a NanoDrop™ 2000 spectrophotometer (Thermo Fisher Scientific), considering 260/280 and 260/230 ratios. RNA integrity was verified through 1% agarose gel electrophoresis. Samples were stored at -80 °C until subsequent analyses. Complementary DNA (cDNA) was generated from 500 ng of total RNA per sample using the High-Capacity cDNA Reverse Transcription Kit (Thermo Fisher Scientific), according to the manufacturer’s instructions.

### Gene expression assays

Relative gene expression was measured through real-time PCR with TaqMan fluorogenic probes (Thermo Fisher Scientific) on an ABI 7500 Real-Time PCR System (Applied Biosystems). Reactions were performed over 40 amplification cycles, in technical duplicates. If cycle quantification values between replicates differed by more than one cycle, reactions were repeated in triplicate, and the two closest values were selected. Target genes included *PDCD1* (Hs01550088_m1), *TIGIT* (Hs00545087_m1), and *HAVCR2* (Hs00958618_m1). Additionally, *GAPDH* (Hs02786624_g1), *ACTB* (Hs03023943_g1), and *RPLP0* (Hs0s00420895_gH) were used as reference genes. Reference gene stability was validated using the geNorm algorithm. Gene expression levels were normalized to the geometric mean of the relative quantities of the reference genes, with relative quantification (relative gene expression) and fold change (FC) values calculated using the 2^−ΔΔCq^ method, following the recommendations of the MIQE 2.0 guidelines for quantitative real-time PCR experiments [25].

### Immunophenotyping

Cell viability, consistently exceeding 95%, was assessed using the Trypan blue exclusion method prior to staining. PBMC were stained with fluorochrome-conjugated monoclonal antibodies for 20 min at room temperature and protected from light. After staining, cells were washed with FACS buffer, fixed with 4% PBS-formaldehyde, and analyzed using an Accuri C6 Flow Cytometer (BD Biosciences). Individual fluorescence compensation was performed for each fluorochrome using single-stained controls, and fluorescence-minus-one (FMO) controls were used to accurately define gating boundaries for each marker. A total of 100,000 events were acquired within the lymphocyte gate for each sample to identify specific CD4 + T cells subsets: recent thymic emigrant (RTE) CD4 + T cells (CD4 + CD45RA+ CD31+), naive CD4 + T cells (CD4 + CD45RA+ CD62L+), central memory CD4 + T cells (CD4 + CD45RA- CD62L+), effector memory CD4 + T cells (CD4 + CD45RA- CD62L-), and effector CD4 + T cells (CD4 + CD45RA+ CD62L-). The fluorescent monoclonal antibodies used included APC-CD4 (EDU-2), BB515-CD4 (RPA T4), PE-CD31 (WM59), PerCP-Cy5.5-CD45RA (HI100), and APC-CD62L (DREG-56), all sourced from BD Biosciences and BioAlbra. The cells were stained for flow cytometry immediately after purification, without cryopreservation. Data was analyzed using FlowJo software, version 10. All subset frequencies are therefore expressed as a percentage of total CD4 + T cells.

### Statistical analysis and graphical representations

Fisher’s exact and Chi-squared tests were used to assess categorical variables. Numerical variables were initially screened for outliers using the ROUT test (Q = 1%), and identified outliers were removed prior to subsequent analyses. The number of outliers removed per variable and group is detailed in Supplementary Table 1. The Shapiro-Wilk test was used to evaluate the Gaussian distribution of numerical variables. Variables with a normal distribution were compared between groups using Student’s t-test and are exhibited as mean ± standard deviation. Meanwhile, for variables that did not follow a normal distribution, the Mann-Whitney test was applied, and the results were presented by the median and interquartile range. Moreover, graphical representations for variables with a normal distribution were displayed as bar plots, representing the mean with standard deviation; whereas non-normally distributed variables were displayed using box-and-whisker plots, representing the median and interquartile range. Correlation coefficients for normal variables were calculated using Pearson’s correlation test, while non-normal variables were calculated using Spearman’s correlation test. The strengths of the correlations were categorized as none (*r* = 0.00), very weak (*r* = 0.01–0.19), weak (*r* = 0.20–0.39), moderate (*r* = 0.40–0.69), strong (*r* = 0.70–0.89), very strong (*r* = 0.90–0.99), and perfect (*r* = 1) according to Schober et al. (2018) [26]. To account for multiple testing across gene expression comparisons, CD4 + T cell subset comparisons, and the correlations, the Benjamini-Hochberg false discovery rate (FDR) procedure was applied within each family, and adjusted p-values (q) were determined and reported to confirm the statistical significance. Receiver operating characteristic (ROC) curve analyses were conducted to assess the discriminatory performance of selected variables in distinguishing INR from IR. The area under the curve (AUC) was used as a measure of accuracy, 95% confidence intervals (95% CI) were also evaluated, with values indicating no discrimination (< 0.70), acceptable (0.71–0.79), excellent (0.8–0.9), and outstanding discriminatory ability (> 0.9) according to criteria adapted from Mandrekar (2010) [27]. Effect sizes for group comparisons involving normally distributed variables were calculated using Cohen’s d, with values interpreted as small (*d* = 0.20–0.49), medium (*d* = 0.50–0.79), and large (*d* ≥ 0.80) adapted from Cohen [28]. For the non-normally distributed variables, effect size was expressed as the rank-biserial correlation coefficient, with values interpreted as small (0.10–0.29), medium (0.30–0.49), and large (≥ 0.50). Post-hoc power analysis was performed based on the observed effect sizes and group sample sizes to contextualize the statistical power achieved for each comparison. The statistical significance level (α) was set at 0.05 for all tests. Statistical analyses and graphical representations were performed using GraphPad Prism (version 8.0.1) and RStudio (version 4.5.1).

## Results

The study included 36 ART-treated male individuals (28 IR and 8 INR). Analysis of the medical records demonstrated that INR exhibited significantly lower pre-ART CD4 + T cell counts/µL compared to IR (236.6 ± 147.2 vs. 449.4 ± 238.3; *p* = 0.017) and after 18 months of treatment (239 ± 158.0 vs. 710.8 ± 211.1; *p* = 0.0001), as well as a lower CD4+ (%) (16.02 ± 6.13 vs. 34.04 ± 10.33; *p* = 0.0006) and CD4/CD8 ratio (0.35 ± 0.14 vs. 0.99 ± 0.59; *p* = 0.0159) after 18 months of ART. Other clinical variables, including age (40.75 ± 11.39 vs. 32.93 ± 10.98 years; *p* = 0.095), therapeutic regimens (*p* = 0.779), and CD8 + T cell absolute count (746.4 ± 309.4 vs. 844.8 ± 487.3 cells/µL; *p* = 0.628) and percentage (45.03 ± 7.42 vs. 37.86 ± 13.69; *p* = 0.2077) after 18 months of ART, did not differ significantly between groups (Table 1). Pre-treatment plasma viral load also did not differ significantly between INR and IR (4.0 ± 0.58 vs. 4.3 ± 0.20 log₁₀ copies/mL; *p* = 0.537), indicating that groups were comparable in initial viral burden.

**Table 1:** Immunological response-based clinical variables of PLHIV during ART

Clinical variable		INR (*n* = 8)	IR (*n* = 28)	*p*-value
Age (years old) at ART start date*		40.75 ± 11.39	32.93 ± 10.98	0.0950
Time (weeks) until ART starting post-diagnosis**		6.0 (2.0-10.7)	6.5 (2.0–9.0)	0.7570
	TDF+3TC + EFZ	2 (25.0)	10 (37.1)	
Detailed ART regimens, NRTI + 3TC + (third option)	TDF+3TC + DTG	5 (62.5)	15 (55.5)	0.7790^a^
	TDF+3TC + ATV/r	1 (12.5)	2 (7.4)	
	2 NRTI + NNRTI	2 (25.0)	10 (37.1)	
ART regimens, stratified by classes	2 NRTI + INI	5 (62.5)	15 (55.5)	0.7790^a^
	2 NRTI + PI/r	1 (12.5)	2 (7.4)	
Pre-treatment PVL (log_10_ RNA copies/mL)*		4.0 ± 0.58	4.3 ± 0.20	0.5370
Pre-treatment CD4 + T-cell count (cells/µL)*		236.6 ± 147.2	449.4 ± 238.3	0.0170
CD4 + T cell count after 18 months of ART (cells/µL)*		239 ± 158.0	710.8 ± 211.1	0.0001
CD8 + T cell count after 18 months of ART (cells/µL)*		746.4 ± 309.4	844.8 ± 487.3	0.6280
CD4+ (%) after 18 months of ART		16.02 ± 6.13	34.04 ± 10.33	0.0006
CD8+ (%) after 18 months of ART*		45.03 ± 7.42	37.86 ± 13.69	0.2077
CD4/CD8 ratio after 18 months of ART*		0.35 ± 0.14	0.99 ± 0.59	0.0159

* t-test (Shapiro-Wilk test: >0.05), values displayed as mean ± standard deviation

**Wilcoxon-Mann-Whitney test (Shapiro-Wilk: <0.05), values displayed as median (IQR)

^a^ Chi-squared test

*3TC* lamivudine, *ART* antiretroviral therapy, *ATV/r* ritonavir-boosted atazanavir, *DTG* dolutegravir, *EFZ* efavirenz, *INI* integrase inhibitor, *INR* immunological non-responders, *IR* immunological responders, *IQR* interquartile range, *NNRTI* non–nucleoside reverse transcriptase inhibitor, *NRTI* nucleoside reverse transcriptase inhibitor, *PI/r* ritonavir-boosted protease inhibitor, *PVL* plasma viral load, *SE* standard error, *TDF* tenofovir

**Fig. 1:**
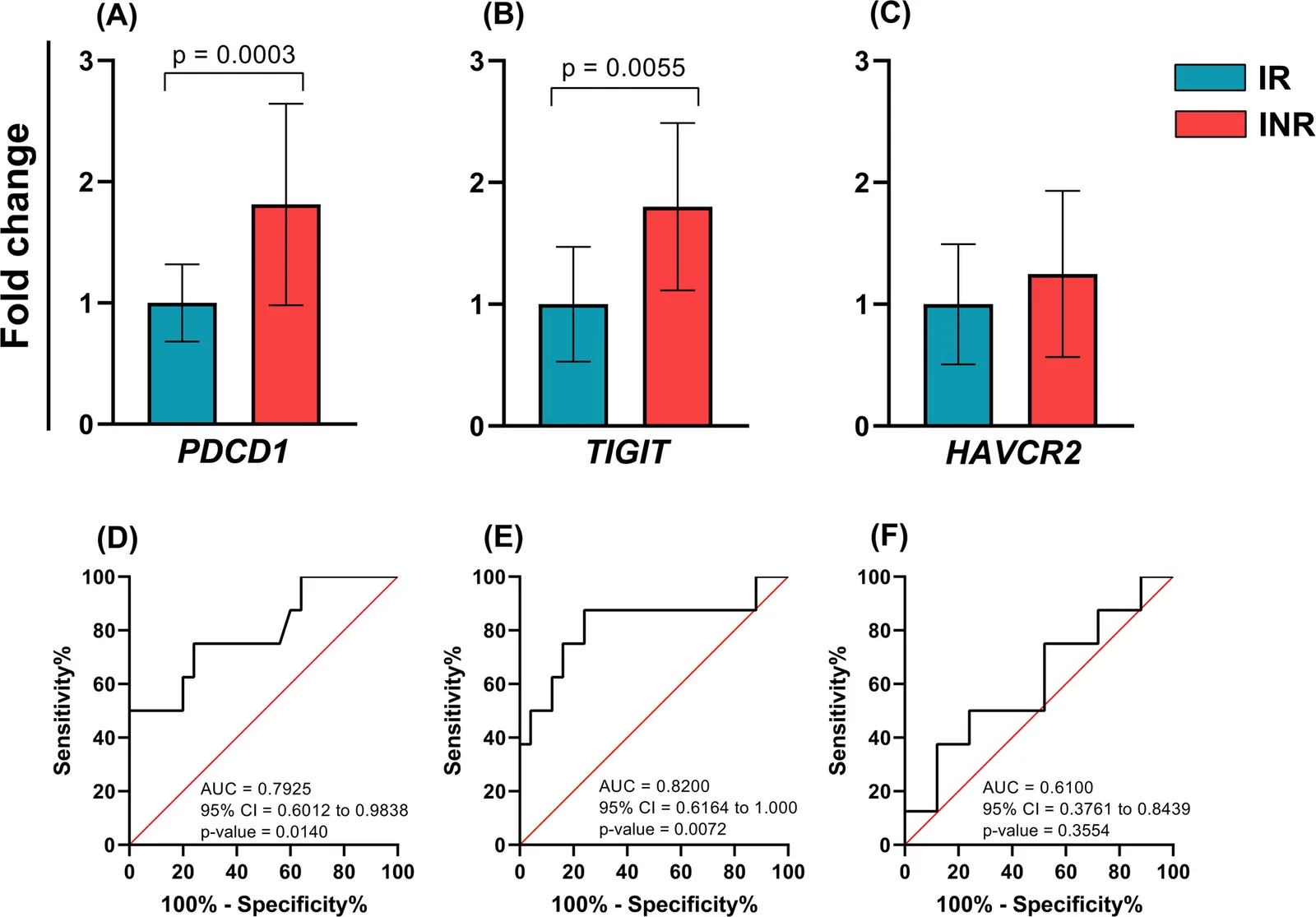
Differential gene expression and ROC analysis of the cellular exhaustion genes in IR vs. INR: (**A**) *PDCD1* expression (FC = 1.81; *p* = 0.0003); (**B**) *TIGIT* expression (FC = 1.80; *p* = 0.0055); (**C**) *HAVCR2* expression (FC = 1.25; *p* = 0.3695); (**D**) *PDCD1* ROC curve (AUC = 0.7925; *p* = 0.0140; 95% CI = 0.6012 to 0.9838); (**E**) *TIGIT* ROC curve (AUC = 0.8200; *p* = 0.0072; 95% CI = 0.6164 to 1.000); (**F**) *HAVCR2* ROC curve (AUC = 0.6100; *p* = 0.3554; 95% CI = 0.3761 to 0.8439)

Regarding gene expression, the INR group exhibited significantly higher expressions of cellular exhaustion markers *PDCD1* (FC = 1.81; *p* = 0.0003; Fig. 1A) and *TIGIT* (FC = 1.80; *p* = 0.0055; Fig. 1B) than the IR group. No significant difference in *HAVCR2* expression was observed between groups (FC = 1.25; *p* = 0.3695; Fig. 1C). Consistently, ROC curve analysis revealed that *PDCD1* (AUC = 0.7925; *p* = 0.0140; 95% CI = 0.6012 to 0.9838; Fig. 1D) and *TIGIT* (AUC = 0.8200; *p* = 0.0072; 95% CI = 0.6164 to 1.000; Fig. 1E) exhibited, respectively, an acceptable and excellent discriminatory ability to distinguish INR from IR. Meanwhile, *HAVCR2* showed poor performance (AUC = 0.6100; *p* = 0.3554; 95% CI = 0.3761 to 0.8439; Fig. 1F).

**Fig2.**
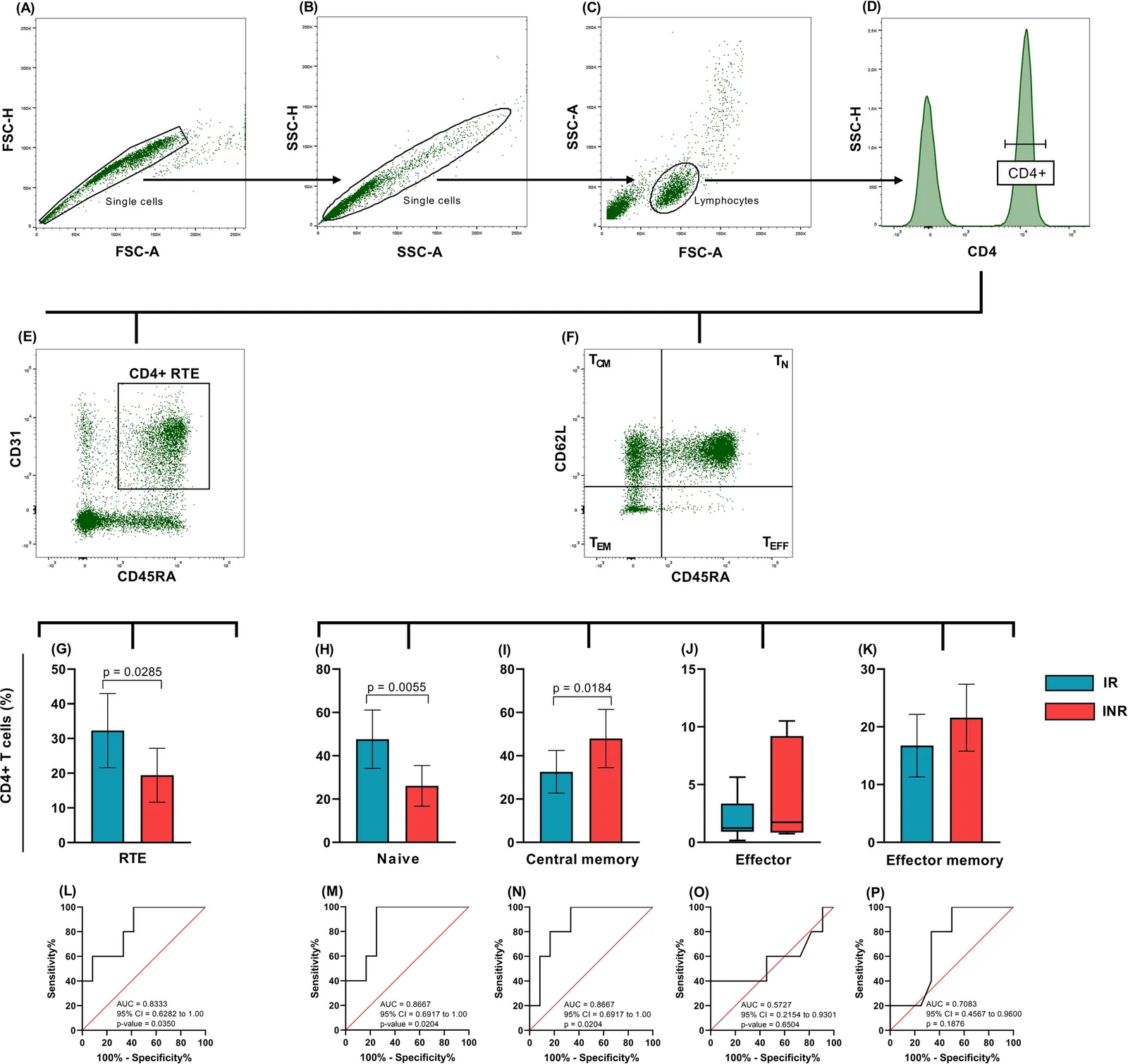
Evaluation of the frequencies of CD4 + T cell subsets among ART-treated PLHIV after 18 months on ART according to immunological response, as displayed in the representative gating strategy (**A**-**F**) and CD4 + T cell subset frequencies (**G**-**K**). Comparisons of frequencies of CD4 + T cell subsets (INR vs. IR): (**G**) RTE (19.42 ± 7.76 vs. 32.29 ± 10.68; *p* = 0.0285); (**H**) Naive (26.10 ± 9.41 vs. 47.65 ± 13.45; *p* = 0.0194); (**I**) Central memory (47.94 ± 13.42 vs. 32.58 ± 9.89; *p* = 0.0184); (**J**) Effector (1.74 [0.84–9.21] vs. 1.24 [0.92–3.35]; *p* = 0.1550); (**K**) Effector memory (21.58 ± 5.80 vs. 16.76 ± 5.42; *p* = 0.1216). ROC curves for each subset: (**L**) RTE (AUC = 0.8333; *p* = 0.0350; 95% CI = 0.6282 to 1.00); (**M**) Naive (AUC = 0.8667; *p* = 0.0204; 95% CI = 0.6917 to 1.00); (**N**) Central memory (AUC = 0.8667; *p* = 0.0204; 95% CI = 0.6917 to 1.00); (**O**) Effector (AUC = 0.5725; *p* = 0.6504; 95% CI = 0.2154 to 0.9301); (**P**) Effector memory (AUC = 0.7083; *p* = 0.1876; 95% CI = 0.4567 to 0.9600). AUC: area under the curve; 95% CI: 95% confidence interval; RTE: recent thymic emigrants; T_N_: naive CD4 + T cell: T_CM_: central memory CD4 + T cell; T_EM_: effector memory CD4 + T cell; T_EFF_: effector CD4 + T cell

We evaluated the frequencies of CD4 + T cell subsets among PLHIV after 18 months on ART, as shown in the representative gating strategy (Figs. 2A-F). Comparative analysis of CD4 + T cell subsets frequencies revealed that INR had significantly lower frequencies of RTE CD4 + T cells (19.42 ± 7.76 vs. 32.29 ± 10.68; *p* = 0.0285; Fig. 2G) and naive CD4 + T cells (26.10 ± 9.41 vs. 47.65 ± 13.45; *p* = 0.0194; Fig. 2H) compared to IR. Conversely, INR exhibited a higher frequency of central memory CD4 + T cells compared to IR (47.94 ± 13.42 vs. 32.58 ± 9.89; *p* = 0.0184; Fig. 2I). No statistically significant differences were observed between INR and IR for effector (1.74 [0.84–9.21] vs. 1.24 [0.92–3.35]; *p* = 0.1550; Fig. 2J) or effector memory (21.58 ± 5.80 vs. 16.76 ± 5.42; *p* = 0.1216; Fig. 2K) CD4 + T cell frequencies. Supporting these results, ROC curve analyses demonstrated that RTE (AUC = 0.8333; *p* = 0.0350; 95% CI = 0.6282 to 1.00; Fig. 2L), naive (AUC = 0.8667; *p* = 0.0204; 95% CI = 0.6917 to 1.00; Fig. 2M), and central memory (AUC = 0.8667; *p* = 0.0204; 95% CI = 0.6917 to 1.00; Fig. 2N) CD4 + T cells exhibited an excellent discriminatory ability between INR and IR. Effector (AUC = 0.5725; *p* = 0.6504; 95% CI = 0.2154 to 0.9301; Fig. 2O) indicated no statistically significant discrimination ability between the groups. Meanwhile, effector memory CD4 + T cells yielded a point estimate within the acceptable discriminatory range, but this did not reach statistical significance (AUC = 0.7083; *p* = 0.1876; 95% CI = 0.4567 to 0.9600; Fig. 2P).

Effect sizes and post-hoc power for all group comparisons is summarized in Supplementary Table 2. Post-hoc power analysis based on the observed effect sizes showed that comparisons with large effects, including RTE, naive, and central memory CD4 + T cell frequencies; as well as *PDCD1* and *TIGIT* expression achieved moderate-to-high power. Meanwhile, comparisons with smaller effects, such as *HAVCR2* expression and effector memory CD4 + T cells, showed lower power.

**Fig. 3:**
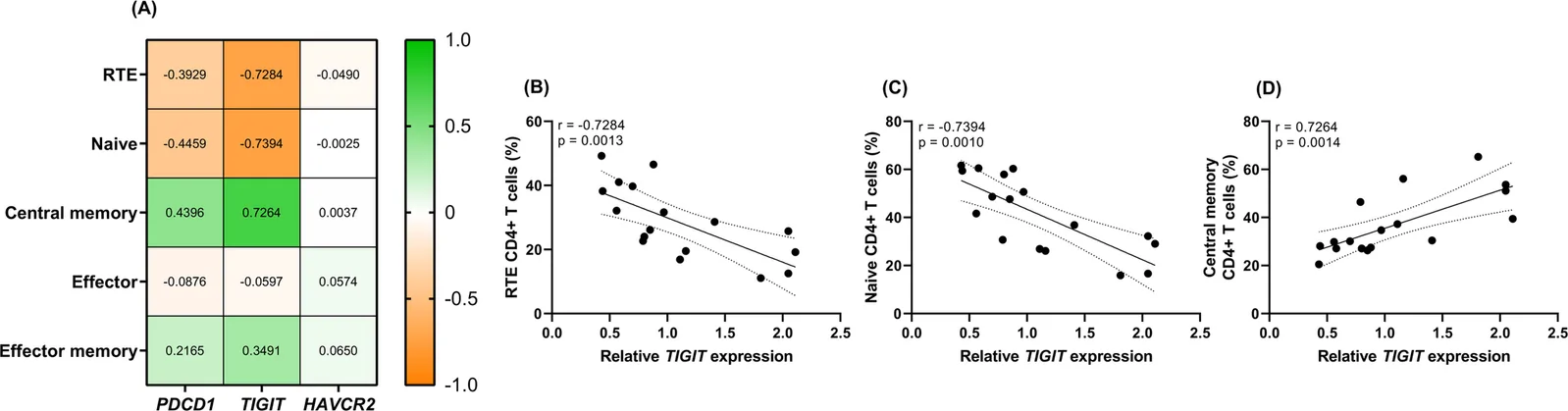
Correlation between relative expression of cellular exhaustion genes and CD4 + T cell subsets frequencies. (**A**) Correlation matrix for the genes (*PDCD1*, *TIGIT*, and *HAVCR2*) and CD4 + T cell subsets (RTE, naive, central memory, effector, and effector memory); (**B**) Correlation between *TIGIT* expression and frequency of RTE CD4 + T cell (*r* = -0.7284; *p* = 0.0013); (**C**) Correlation between *TIGIT* expression and frequency of naive CD4 + T cell (*r* = -0.7394; *p* = 0.0010); (**D**) Correlation between *TIGIT* expression and frequency of central memory CD4 + T cell (*r* = 0.7264; *p* = 0.0014). RTE: recent thymic emigrants

Following the comparisons between IR and INR groups, correlation analyses were performed using the entire cohort of male individuals living with HIV on ART for 18 months with undetectable viral loads, aiming to elucidate the impact of cellular exhaustion gene expression levels on the frequencies of CD4 + T cell subsets. Therefore, a matrix was generated to assess the correlations between the relative gene expression of cellular exhaustion markers and the frequencies of CD4 + T cell subsets (Fig. 3A). *TIGIT* expression demonstrated strong and statistically significant inverse correlations with the frequencies of RTE CD4 + T cells (*r* = -0.7284; *p* = 0.0013; Fig. 3B) and naive (*r* = -0.7394; *p* = 0.0010; Fig. 3C) CD4 + T cells. In contrast, *TIGIT* expression was strongly and directly correlated with the frequency of central memory CD4 + T cells (*r* = 0.7264; *p* = 0.0014; Fig. 3D). *PDCD1* and *HAVCR2* did not demonstrate statistically significant correlations (more details in Supplementary Fig. 1).

Stratified analysis confirmed the same direction of correlation between *TIGIT* expression and CD4 + T cell subsets observed in the pooled cohort (negative for RTE and naive, and positive for central memory) in both IR and INR groups (Supplementary Fig. 2). These correlations remained statistically significant in the IR group, whereas in INR the same pattern did not reach statistical significance, likely reflecting the smaller sample size rather than a divergent underlying relationship.

Importantly, after Benjamini-Hochberg correction within each family of comparisons, all significant findings remained significant (q < 0.05): *PDCD1* (q = 0.0009) and *TIGIT* (q = 0.0083) expression; RTE (q = 0.0475), naive (q = 0.0275), and central memory (q = 0.0460) CD4 + T cell frequencies (Supplementary Table 3).

## Discussion

Advances in ART have significantly reduced morbidity and mortality among PLHIV. Nevertheless, important challenges remain to be completely elucidated, including the mechanisms underlying immunological nonresponse and T cell exhaustion, both of which are critical determinants of long-term health outcomes and quality of life of ART-treated PLHIV [9, 20]. Previous evidence suggests that ART only partially downregulates the expression of ICR on the surface of T cells [29, 30]. Consequently, even individuals with sustained viral suppression exhibit higher ICR expression compared to healthy controls, whereas INR often exhibit higher levels of these receptors than IR [31, 32].

Persistent and elevated immune activation in ART-treated PLHIV is an important mechanism that contributes to immunological nonresponse and T cell exhaustion [33, 34]. Our results revealed an upregulation of *PDCD1* and *TIGIT* in INR compared to IR. ROC curve analysis confirmed the discriminative capacity of *PDCD1* and *TIGIT* expression reliably distinguishing INR from IR. Interestingly, the levels of the receptors PD-1 and TIGIT on the surface of CD4 + T cells have been directly correlated with disease progression and inversely with CD4 + T cell counts, characteristics that usually define the immunological nonresponse [35, 36]. Moreover, T cells displaying higher ICR expression tend to harbor larger latent viral reservoirs, leading to residual viral replication and persistent immune activation. This ongoing activation promotes T cell exhaustion and could further contribute to immunological nonresponse [37–39].

Baseline viral load, a main driver of persistent immune activation, and a potential confounder of immunological nonresponse did not differ between groups [40]. Therefore, the elevated *PDCD1* and *TIGIT* expression observed in INR is unlikely to reflect differences in pre-treatment viral burden, supporting the interpretation that incomplete immune reconstitution is influenced by T cell exhaustion rather than by baseline viral load. Furthermore, consistent with an exhaustion-driven phenotype, INR also exhibited a markedly lower CD4/CD8 ratio than IR, a well-established surrogate of persistent immune activation, senescence, and T cell exhaustion that reinforces the association between incomplete immune reconstitution and the exhaustion markers evaluated [41, 42].

This study also demonstrated that INR presented lower frequencies of RTE and naive CD4 + T cells after 18 months of ART compared with IR. This finding may suggest a reduced thymic output or impaired renewal of CD4 + T cells in INR, consistent with previous reports, including our group’s results in a distinct population, identifying reduced frequencies of RTE and naive cells as risk factors for immunological nonresponse [34, 43, 44]. In contrast, INR exhibited higher frequencies of central memory CD4 + T cells compared to IR. Although central memory cells are important in maintaining immune homeostasis and present a strong proliferative capacity, during HIV infection, these cells are major contributors to the maintenance of residual viral replication. Thus, contributing to persistent cellular depletions even on viral suppression, which could lead to immunological nonresponse [45, 46].

Our correlation matrix revealed a strong inverse correlation between *TIGIT* expression and the frequencies of RTE and naive CD4 + T cells. This is particularly relevant, as exhausted T cells exhibit reduced proliferative and effector capacity, along with decreased secretion of IL-2 and IL-7, leading to impaired lymphocyte renewal and compromised immune responses [47, 48]. Meanwhile, a strong direct correlation was observed between *TIGIT* expression and the frequency of central memory CD4 + T cells. This correlation suggests that the expansion of this subset may be related to cellular exhaustion, possibly because of persistent immune activation, ultimately limiting immune reconstitution of ART-treated PLHIV [31, 49, 50].

Recent evidence has demonstrated that the upregulation of the TIGIT receptor on the surface of T cells disrupts CD226-mediated co-stimulation, compromising the immune responses of natural killer and T cells [31, 51]. Beyond the HIV infection, there have been associations between the upregulation of the TIGIT receptor in T cells during bacterial infection [52]. Additionally, TIGIT may promote CD8 + T cell exhaustion during colorectal cancer, as well as be used to predict poor prognosis of these individuals [53]. Several studies have also indicated that TIGIT blockade can reverse the cellular exhaustion phenotype, restore antiviral activity, and promote the production of homeostatic cytokines [36, 54, 55]. Although evidence suggests the correlation between the levels of TIGIT on the cell surface with dysregulation in immune responses (reduced production of IL-2 and IFN-γ) and chronic inflammation (high levels of IL-6, CD38, and sCD14), there are fewer studies regarding *TIGIT* gene expression and its impact on immune responses, especially in PLHIV [49, 56–58].

The lack of significant *HAVCR2* upregulation in INR, unlike *PDCD1* and *TIGIT*, may reflect a biologically meaningful finding rather than simply a negative result. Unlike our flow cytometry analyses, which targeted CD4 + T cell subsets specifically, gene expression was assessed in whole PBMC. Since TIM-3 is preferentially expressed on CD8 + T cells and innate populations, a specific CD4 + T cell *HAVCR2* upregulation may have been diluted by these other cell types [59, 60]. Moreover, TIM-3 kinetics during ART can also differ from those of PD-1 and TIGIT, with partial normalization reported within the first year of treatment, and its surface expression is further shaped by ligand-dependent post-transcriptional regulation not necessarily reflected at the transcript level [60, 61].

This study has certain limitations that should be acknowledged. The sample size was limited and restricted to male individuals, primarily due to challenges in recruiting women for the INR group, as females are less likely to experience immunological nonresponse [62, 63]. The combination of a naturally uncommon phenotype with the rigorous inclusion requirements substantially narrowed the pool of eligible INR participants despite active recruitment. However, the strict eligibility criteria and matching group strategies were important to minimize confounding of gene expression and flow cytometry results by confounding factors. Moreover, a post-hoc power analysis based on the observed effect sizes indicated that comparisons with large effect sizes, such as naive, central memory, and RTE CD4 + T cell frequencies, and *PDCD1* and *TIGIT* expression, achieved moderate-to-high statistical power, whereas comparisons with smaller effect sizes, such as *HAVCR2* expression and effector memory CD4 + T cells, achieved comparatively lower power. This indicates that non-significant findings for these variables should be interpreted with caution, as they may reflect insufficient power to detect smaller effects rather than a true absence of biological difference.

A further limitation concerns the cellular source and analytical level of our gene expression data: RNA was extracted from bulk PBMC rather than sorted CD4 + T cells, and monocyte and natural killer cell frequencies were not assessed. Although PD-1 and TIGIT are predominantly T cell exhaustion markers, we cannot fully exclude a contribution of shifts in PBMC composition to the observed transcript differences. Additionally, as we did not perform protein-level or functional validation, and transcript abundance can diverge from surface protein expression due to post-transcriptional regulation and receptor shedding, comparisons with studies reporting receptor surface density remain indirect [64, 65]. Importantly, our design is associative rather than causal: whether TIGIT-expressing CD4 + T cells directly contribute to the failure of CD4 + T cell recovery, or instead represent a marker of an underlying dysfunctional state, remains to be established through functional assays. Our findings should therefore be interpreted as evidence of altered *PDCD1* and *TIGIT* transcription in bulk PBMC, rather than as direct evidence of protein-level T cell exhaustion, warranting validation in sorted CD4 + T cells with protein-level and functional assays.

Despite these constraints, the study’s robustness is maintained through meticulous matching of groups for variables such as ART duration, therapeutic regimens, and absence of clinical confounders, which is critical for minimizing biological variability in gene expression and flow cytometry analyses. Furthermore, the analysis of clinical variables corroborates established findings, such as the association between low baseline CD4 + T cell counts and an increased risk of immunological nonresponse [66–68]. Additionally, this study offers novel insights by investigating cellular exhaustion in the context of immune reconstitution, an underexplored area, particularly at the transcriptional level. These findings offer valuable insights into the mechanisms underlying incomplete immune reconstitution in ART-treated PLHIV.

## Conclusion

In conclusion, our findings suggest that immunological nonresponse in ART-treated individuals may be linked to the upregulation of genes associated with T cell exhaustion and to disruptions in the frequencies of CD4 + T cell subsets. The correlations observed between *TIGIT* expression and CD4 + T cell frequencies reinforce the possibility that exhaustion-mediated disturbances in T cell homeostasis contribute to immunological nonresponse. Taking together, these results may help refine the understanding of the biological mechanisms underlying poor CD4 + T cell reconstitution and highlight potential molecular targets for improving clinical monitoring and guiding future therapeutic strategies. These conclusions are drawn at the transcriptional level in bulk PBMC and should be regarded as hypothesis-generating, therefore, protein-level and functional validation in sorted CD4 + T cells are needed before these findings can be extrapolated to T cell exhaustion at the cellular/functional level.

## Supplementary Information

Below is the link to the electronic supplementary material.

Supplementary Material 1 (DOCX 460 KB)

Supplementary Figure 1 (PNG 192 KB)

High Resolution Image (EPS 977 KB)

Supplementary Figure 2 (PNG 155 KB)

High Resolution Image (EPS 76.3 KB)

## Data Availability

No datasets were generated or analysed during the current study.
