# Supplementary material for: Upregulation of cellular exhaustion-related genes and CD4 + T cell disturbances in immunological non-responders on antiretroviral therapy
Source: Immunol Res. 2026 Oct 9;74(1):125. doi: 10.1007/s12026-026-09859-y (PMC13652942; doi:10.1007/s12026-026-09859-y)
Supplement: Supplementary file 1 [file 12026_2026_9859_MOESM1_ESM.docx]

**Supplementary Table 1**. Number of outliers identified and excluded per variable and group using the ROUT method (Q = 1%) prior to statistical analysis.

| **Variable** | **IR** | **INR** |
| --- | --- | --- |
| Age (years old) at ART start date | 0 | 0 |
| Time (weeks) until ART starting post-diagnosis | 0 | 0 |
| Pre-treatment PVL (log_10_ RNA copies/mL) | 0 | 0 |
| Pre-treatment CD4+ T-cell count (cells/µL) | 0 | 0 |
| CD4+ T cell count after 18 months of ART (cells/µL) | 1 | 1 |
| CD8+ T cell count after 18 months of ART (cells/µL) | 0 | 0 |
| CD4+ (%) after 18 months of ART | 0 | 1 |
| CD8+ (%) after 18 months of ART | 0 | 0 |
| CD4/CD8 ratio after 18 months of ART | 1 | 1 |
| PDCD1 expression | 3 | 0 |
| TIGIT expression | 2 | 0 |
| HAVCR2 expression | 3 | 0 |
| RTE CD4+ T cell frequencies (%) | 0 | 0 |
| Naive CD4+ T cell frequencies (%) | 0 | 0 |
| Central memory CD4+ T cell frequencies (%) | 0 | 0 |
| Effector CD4+ T cell frequencies (%) | 0 | 0 |
| Effector memory CD4+ T cell frequencies (%) | 1 | 0 |

IR: immunological responders; INR: immunological non-responders; RTE: recent thymic emigrant

**Supplementary Table 2.** Effect sizes and post-hoc statistical power for group comparisons

| **Variable** | **Statistical test** | **Effect size (*d*)** | **Magnitude** | **Pos-hoc power** |
| --- | --- | --- | --- | --- |
| PDCD1 expression | t-test | 1,68 | Large | 98% |
| TIGIT expression | t-test | 1,53 | Large | 96% |
| HAVCR2 expression | t-test | 0,44 | Small | 17% |
| RTE CD4+ T cells (%) | t-test | 1,29 | Large | 62% |
| Naive CD4+ T cells (%) | t-test | 1,72 | Large | 87% |
| Central memory CD4+ T cells (%) | t-test | 1,40 | Large | 69% |
| Effector CD4+ T cells (%) | Mann-Whitney | 0,36 | Moderate | - |
| Effector memory CD4+ T cells (%) | t-test | 0,87 | Large | 31% |

**Supplementary figure 1**. Correlations between expression of cellular exhaustion genes and frequencies of CD4+ T cells subsets

**Supplementary Figure 2**. Correlation heatmaps between cellular exhaustion gene expression and CD4+ T cell subset frequencies, stratified by group: (A) Immunological Responders (B) Immunological Non-Responders.

**Supplementary Table 3**. Multiple comparison correction (Benjamini-Hochberg FDR) for gene and CD4+ T cell subset frequencies expression between IR and INR groups.

| **Variable** | **p-value** | **q (BH-FDR)** |
| --- | --- | --- |
| *PDCD1* expression | 0,0003 | 0,0009 |
| *TIGIT* expression | 0,0055 | 0,0083 |
| *HAVCR2* expression | 0,3695 | 0,3695 |
| RTE (%) | 0,0285 | 0,0475 |
| Naïve (%) | 0,0055 | 0,0275 |
| Central memory (%) | 0,0184 | 0,0460 |
| Effector (%) | 0,1550 | 0,1550 |
| Effector memory (%) | 0,1216 | 0,1520 |

BH-FDR: Benjamini-Hochberg false discovery rate; IR: immunological responders; INR: immunological non-responders; RTE: recent thymic emigrant
